# Yarn-Based Aligned Recycled Carbon Fiber/PA11 Composites: Yarn Development, Coupon Characterization, Initial Structural Demonstration, and Screening Life-Cycle Assessment

**DOI:** 10.3390/polym18212658

**Published:** 2026-10-27

**Authors:** Christian Brauner, Delal Arslan, Julian Kupski, Florian Givel, Mohammad Hajikazemi, Miriam Preinfalck, Stephan Baz, Götz T. Gresser, Jascha Schmied, Sebastian Bühler, Christian Braun

**Affiliations:** 1 Institute of Polymer Engineering, FHNW University of Applied Sciences and Arts Northwestern Switzerland, Klosterzelgstrasse 2, 5210 Windisch, Switzerland; delal.arslan@fhnw.ch (D.A.); julian.kupski@ditf.de (J.K.); florian.givel@fhnw.ch (F.G.); mohammad.hajikazemi@fhnw.ch (M.H.); 2 German Institutes of Textile and Fiber Research Denkendorf (DITF), Körschtalstrasse 26, 73770 Denkendorf, Germanystephan.baz@ditf.de (S.B.); goetz.gresser@ditf.de (G.T.G.); 3 Bionic Composite Technologies AG, Flurhofstrasse 150, 9000 St. Gallen, Switzerland; jascha.schmied@biontec.ch (J.S.); sebastian.buehler@biontec.ch (S.B.); 4 Ensinger Composites Schweiz GmbH, Libernstrasse 24, 8112 Otelfingen, Switzerland; christian.braun@ensingerplastics.com

**Keywords:** hybrid yarn, recycled carbon fiber (rCF), polyamide 11 (PA11), roller carding, tailored fiber placement, compression molding, structural demonstrator, life-cycle assessment

## Abstract

Re-aligned recycled carbon fiber (rCF) yarns provide a complementary architecture to aligned rCF tapes, enabling load-path-oriented reinforcement placement with potential for automated near-net-shape preforming and three-dimensional formability. This study investigates hybrid rCF/polyamide 11 (PA11) wrap yarns produced through roller carding, hollow-spindle wrap spinning, tailored fiber placement (TFP), and consolidation. Two rCF/PA11 yarn formulations with nominal fiber/matrix mass ratios of 72/28 and 80/20 were developed, yielding fiber volume fractions of 54.4 and 58.5 vol.%, respectively. Mechanical characterization focused on UD coupon testing under axial tensile, transverse tensile, and shear loading. For the rCF/PA11 72/28 and 80/20 formulations, longitudinal tensile testing yielded moduli of 57.8 ± 4.2 and 64.3 ± 6.3 GPa and strengths of 920 ± 41 and 815 ± 86 MPa, respectively. The 72/28 material additionally exhibited an in-plane shear modulus of 1.87 ± 0.03 GPa and shear strength of 48.7 ± 1.4 MPa. Relative to the previously reported aligned rCF/PA11 tape laminates at a comparable fiber volume content, albeit with distinct processing machinery, the 72/28 yarn laminate showed higher longitudinal modulus and longitudinal strength but substantially lower transverse and shear properties, suggesting a possible architectural trade-off associated with discrete yarn lanes, helical wrapping, and TFP preforming. A conrod-shaped preliminary structural benchmark manufactured from rCF/PA11 72/28 sustained 11.3 ± 0.5 kN at a mass of 23.0 ± 1.0 g, corresponding to a 71% higher specific load capacity than the aluminum reference. Proof-of-concept thermoforming of three double-curved shell demonstrators confirmed the capability of the yarn-based preforms to form complex three-dimensional geometries, although local fiber alignment defects highlighted the need for improved draping control. A screening life-cycle assessment estimated 0.42 kg CO_2_-eq per rCF/PA11 benchmark part, representing reductions of approximately 63% relative to the virgin-CF/PA12 stretch-broken yarn route and 52% relative to virgin-CF/epoxy routes. The results demonstrate the potential of rCF/PA11 wrap yarns as high-fiber-content textile intermediates for load-path-oriented TFP preforming and subsequent three-dimensional thermoforming.

## 1. Introduction

Carbon-fiber-reinforced polymers provide high specific stiffness and strength but create a growing challenge at manufacturing and end of life, as the high-value carbon fibers are embedded in a polymer matrix that complicates their recovery and reuse. Recent sustainability assessments further emphasize that the energy-intensive production of carbon fibers and the limited recyclability of conventional polymer matrices remain major barriers to achieving circular carbon-fiber composites. Advances in recyclable resin systems, remanufacturing, and the re-alignment of recycled fibers therefore represent promising strategies for improving the circularity and overall sustainability of carbon-fiber composites [1]. Thermal, chemical, and mechanical reclamation routes can recover carbon fibers, yet the resulting material is usually discontinuous and has lost the orientation and textile architecture that originally enabled structural performance [2,3,4,5]. Direct reuse as milled filler or randomly oriented nonwoven reinforcements [6] is technically accessible, but it captures only a small fraction of the fibers’ axial properties. The central remanufacturing challenge is therefore not only fiber recovery but also controlled re-alignment of discontinuous fibers into continuous textile intermediate products such as yarns and tapes.

Aligned discontinuous fiber composites can recover a substantial fraction of the longitudinal performance of continuous-fiber systems when fiber length, orientation, fiber volume fraction, and consolidation are adequately controlled [7,8]. Several alignment routes have been demonstrated [9], including aqueous deposition, centrifugal alignment, and textile carding-based processes followed by tape forming or yarn spinning. Such textile processing routes are particularly attractive for medium-to-long staple fibers because conventional textile equipment can open, blend, orient, and continuously transport the recovered reinforcement. Khurshid et al. [10] converted recycled carbon staple-fiber yarns into unidirectional tapes with more than 90% of fibers within ±10° after stretching and calendaring. More recently, Theiss et al. [11] showed that modified carding combined with subsequent alignment can produce highly oriented rCF tape intermediates for automated placement. Previous multiscale characterization [7] demonstrated that orientation distributions, fiber length, local compaction [12], and architectural asymmetry [13] govern the effective properties of staple-fiber composites.

Hybrid yarns provide a different processing and design space compared to flat tape. The feasibility of converting recycled carbon fibers into continuous yarn intermediates has been demonstrated using several spinning technologies, including flyer spinning, ring spinning, friction spinning, and wrap spinning [14,15]. These processes differ in the way cohesion is introduced into the staple fiber assembly and therefore in the resulting fiber arrangement and yarn structure. For flyer-spun hybrid yarns, Hengstermann et al. [14] showed that permanent yarn twist affects the tensile properties of the resulting UD composites, with increasing twist generally increasing fiber deviation from the yarn axis. More recently, Hasan et al. [15] demonstrated that yarn architecture itself affects yarn compactness, hairiness, resin infiltration, and the distribution of fiber orientations within the resulting composite by comparing friction-spun and wrap-spun rCF yarns. Wrap spinning is particularly attractive for composite applications because yarn cohesion is provided through an external wrapping filament, preserving a largely twist-free and axially aligned staple fiber core. In hollow-spindle wrap spinning, the carbon staple fiber sliver is first drafted to achieve the target linear density and improve fiber alignment, after which a continuous filament is helically wrapped around the core to provide yarn cohesion and handling stability. Akonda et al. [16] demonstrated the feasibility of this approach for carded rCF/PP hybrid yarns. Optionally, false twist can be applied before wrapping; although excessive levels may promote more tortuous fiber paths, moderate false twist can improve temporary core cohesion and yarn processability [17]. Yarn-based textile intermediates are also compatible with weaving, tailored fiber placement (TFP), near-net-shape preforming, and deep drawing of thermoplastic organosheets [18,19,20,21]. This manufacturing flexibility is relevant for complex structures requiring load-path-oriented reinforcement placement and subsequent three-dimensional forming.

Polyamide 11 (PA11) is a predominantly bio-based engineering thermoplastic derived from castor oil and combines thermoplastic recyclability with useful toughness and chemical resistance [22]. Recent work has investigated several rCF/PA11 material systems and reinforcement configurations. Short-rCF PA11 compounds [23] can be injection molded or additively processed, but their performance is limited by fiber attrition, limited fiber alignment, and low fiber volume content. Mechanically recycled aerospace prepreg waste has also been investigated as discontinuous reinforcement in PA11, demonstrating the feasibility of incorporating reclaimed CFRP fragments into bio-based thermoplastic composites [24]. Most directly relevant to the present study, the recently published paper on aligned PA11/rCF tape route [25] demonstrated that textile alignment and consolidation can produce highly aligned rCF thermoplastic laminates with a longitudinal tensile modulus of 48.1 ± 4.6 GPa and a strength of 866 ± 127 MPa at 56.7 vol.% fiber. While aligned tapes provide an efficient approach for planar reinforcement and can also be formed into complex geometries, tailoring reinforcement paths to component-specific load requirements may require additional cutting, placement, or assembly operations. Yarn-based architectures offer a complementary approach by enabling reinforcement to be deposited along predefined load paths using processes such as tailored fiber placement (TFP). This approach has the potential to facilitate load-path-specific reinforcement and near-net-shape preforming, while offering flexibility in the fabrication of three-dimensional preforms. In particular, the use of a single-sided preforming tool in TFP can provide an alternative to tape-based preforming approaches that rely on two-sided tooling. Despite these developments, the potential of rCF yarns as integrated textile intermediates for high-performance bio-based PA11 composites remains insufficiently established. Previous studies have demonstrated rCF yarn formation and individual downstream processing steps, including TFP and thermoforming. However, an integrated assessment linking yarn architecture, fiber alignment, high fiber-volume-content consolidation, structural performance, and three-dimensional near-net-shape manufacturing is still lacking. This motivates the present investigation of rCF/PA11 wrap yarns as continuously processable textile intermediates, with emphasis on high-fiber-volume-fraction consolidation, load-path-oriented TFP preforming and subsequent three-dimensional forming.

The present study investigates a yarn-based processing route for rCF/PA11 composites and evaluates the complete process–property chain with some insights on microstructures. The objectives were to (i) manufacture high-fiber-content rCF/PA11 wrap yarns and TFP preforms; (ii) characterize longitudinal, transverse, and shear properties at coupon level; (iii) place the mechanical performance of the yarn-based laminates in the context of previously reported aligned rCF/PA11 tape results [25]; (iv) perform initial structural and formability assessment via a benchmark geometry and thermoformed demonstrator parts; and (v) conduct a screening life-cycle assessment.

## 2. Materials and Methods

### 2.1. Materials and Overall Process Route

The reinforcement consisted of recovered Toray T700SC-12K-50C carbon staple fibers (Toray Industries, Chuo City, Tokyo, Japan) obtained from production off-cuts with a nominal filament diameter of approximately 7 μm. The recovered fibers had an initial length of approximately 80 mm before opening and carding, which was reduced to approximately 50 mm after carding. The approximate lengths of 80 mm before opening/carding and 50 mm after carding describe the reported processing stages. Quantitative statistical fiber-length distributions after carding and after wrap spinning are not available for the present study. In accordance with Kelly–Tyson shear-lag theory [26], the critical fiber length (l_c_) for carbon fiber/polyamide interfaces typically ranges between 0.5 mm and 1.5 mm. This critical fiber length is a function of fiber diameter and fiber and interface strength properties. Because processing waste of high structural integrity was utilized without significant degradation with respect to fiber or interfacial strength, the mean post-carding staple fiber length of approximately 50 mm remains well above l_c_ (l >> l_c_), maintaining near-optimal stress transfer efficiency during tensile loading.

The thermoplastic matrix was based on Rilsan^®^ PA11 (Arkema SA, Colombes, France) and was introduced in different textile forms, including staple fibers, a wrapping filament, and the TFP stitching yarn. PA11 was selected because its feedstock is predominantly derived from renewable castor oil. The PA11 staple-fiber specification was established based on a preceding full-factorial screening study in which finish content, spinning draw ratio, and single-fiber linear density were varied and evaluated through carding trials. The selected PA11 staple fibers had a nominal linear density of approximately 3 dtex, a cut length of 60 mm, a finish content of approximately 0.4 wt.%, a spinning draw ratio of approximately 1:1.1, and a crimped morphology. In addition to the rCF/PA11 coupon specimens, an rCF/PA6 reference formulation with a nominal fiber/matrix mass ratio of 70/30 was manufactured using the same rCF and identical wrap-spinning parameters, but with PA6 as the matrix fiber. Conventional Grilon^®^ P300 PA6 staple fibers (EMS-GRILTECH, Domat/Ems, Switzerland) with a cut length of 60 mm and a nominal linear density of approximately 3.3 dtex were used as matrix fibers. While coupon-level testing was limited to rCF-based yarn laminates, virgin-CF/PA12, virgin-CF/epoxy RTM, and aluminum AW 6082 references were additionally used for the component-level benchmark.

**Figure 1:**
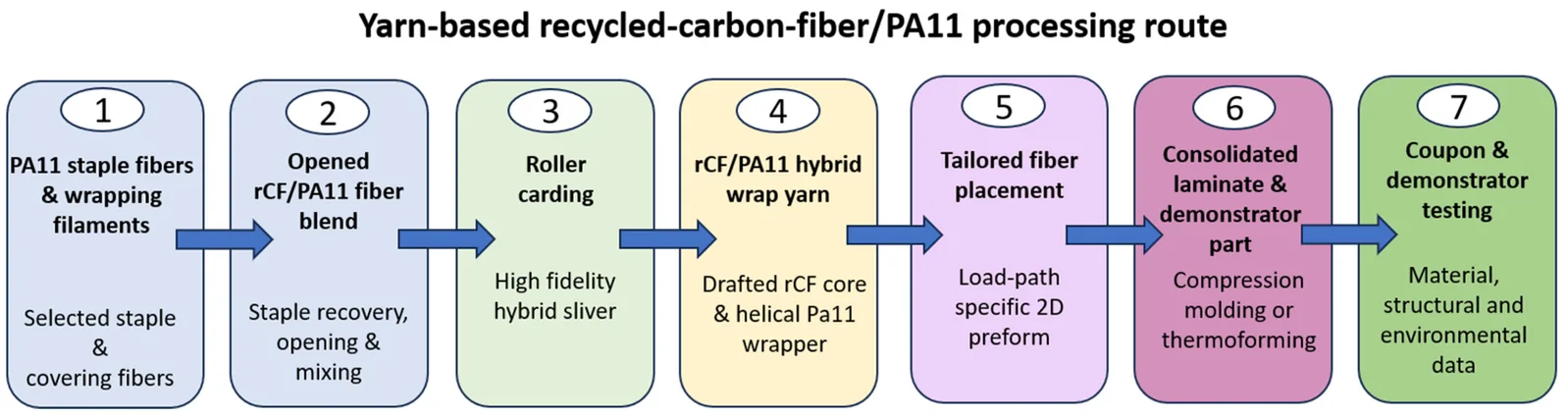
Yarn-based rCF/PA11 processing route from PA11 staple fibers and recovered carbon-staple fibers to card sliver, wrap yarn, TFP preform, consolidated coupons, and structural demonstrators.

The yarn-based route is summarized in Figure 1. PA11 staple fibers were blended with rCF, roller-carded into a preferentially aligned sliver, drafted, and stabilized by hollow-spindle wrap spinning with a continuous PA11 filament. The resulting yarn was placed into flat or load-path-adapted preforms by TFP and consolidated by compression molding. A parallel organosheet route enabled matched-die thermoforming of a double-curved shell demonstrator.

### 2.2. Opening, Blending, Roller Carding, and Wrap-Yarn Spinning

Prior to carding by means of a roller card, the fibers underwent two opening passes using an RE5 opener (DILO Machines GmbH, Eberbach, Germany). In the first pass, only the rCF was opened; in the second pass, the opened rCF was blended with the respective PA11 or PA6 staple fibers.

**Figure 2:**
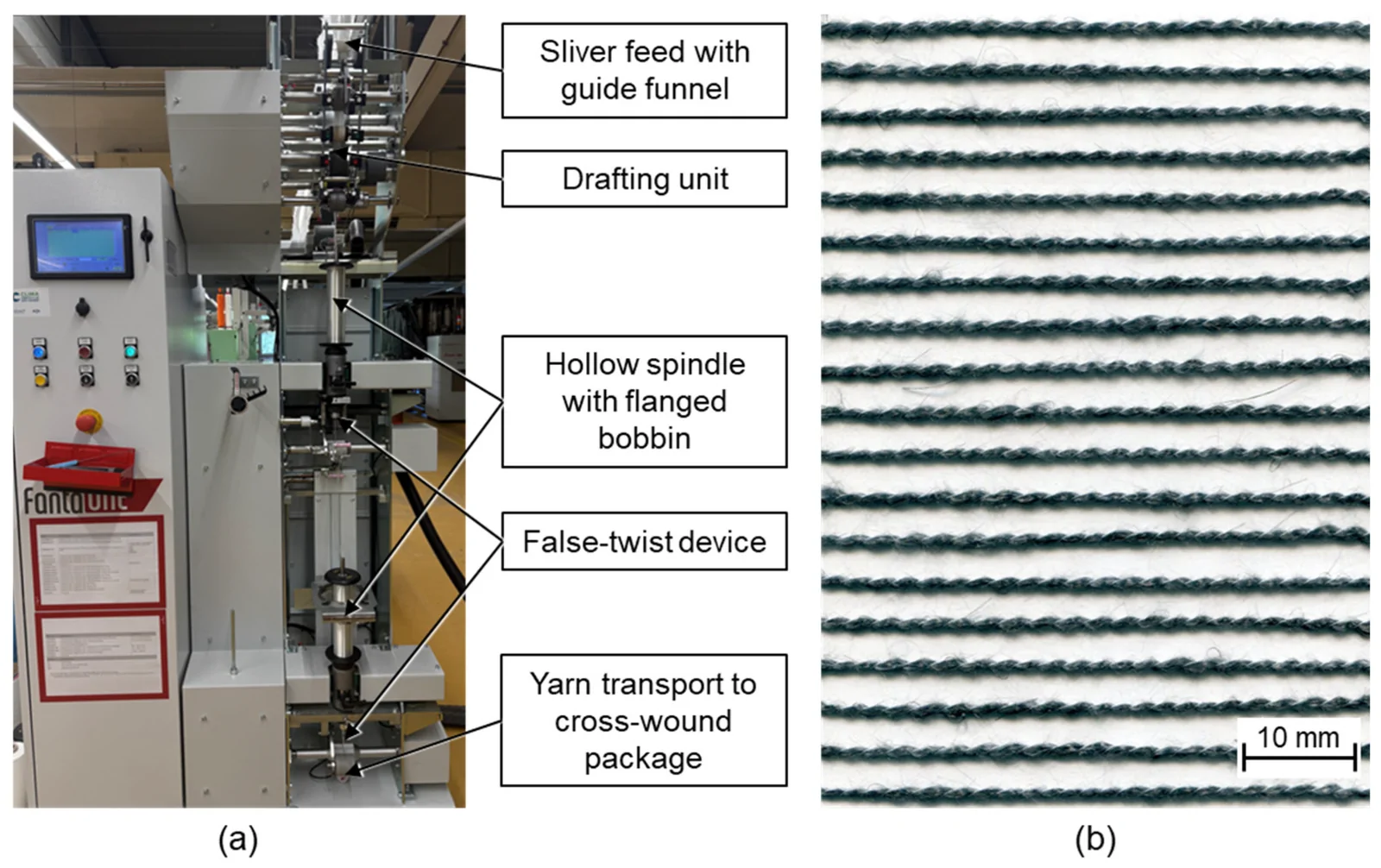
Hollow-spindle wrap-yarn manufacturing: (**a**) Fanta One wrap-spinning machine and (**b**) representative rCF/PA11 hybrid wrap yarn.

The resulting blends were subsequently processed on a 1000 mm wide roller-carding line (Off. Mecc. Pietro Ramella & C. s.a.s., Biella, Italy; Figure 2a). The principal carding parameters are summarized in Table 1. The fiber flocks on the feeding table were fed to the card with a grammage of approximately 250 g/m^2^ and a feeding speed of 0.061 m/min, corresponding to a nominal mass flow of 15.25 g/min related to the width of the roller card with a processing width of 1000 mm. The main cylinder was operated at 193 rpm, while the relative surface speeds of the active carding elements were adjusted according to the ratios given in Table 1. The carded web was condensed into a preferentially aligned sliver with a target linear density of 2.0 ktex for drafting and subsequent wrap-yarn spinning.

The pre-oriented sliver from the roller-carding machine was drafted and subsequently processed by hollow-spindle wrap spinning on a Fanta One spinning machine (Gualchieri & Gualchieri & C. S.r.l., Prato, Italy; Figure 2a). A false-twist device was used upstream of the wrapping zone to provide temporary cohesion to the drafted staple fiber core during yarn formation. The core was subsequently stabilized by helically wrapping it with a continuous thermoplastic filament, resulting in a largely twist-free hybrid wrap yarn. A continuous PA11 filament was used to wrap the rCF/PA11 yarns, whereas a PA6.6 filament was used for the rCF/PA6 reference yarn. A representative rCF/PA11 wrap yarn is shown on the yarn board in Figure 2b. The principal wrap-spinning parameters are summarized in Table 2. Two slivers with a target linear density of approximately 2 ktex each were supplied to the drafting unit, with the total draft adjusted depending on the actual sliver linear density to achieve a target yarn linear density of 800 tex.

The yarn formulations investigated at the coupon level comprised rCF/PA11 72/28 and rCF/PA11 80/20, as well as the rCF/PA6 70/30 reference formulation, where the two numbers denote the fiber and matrix mass percentages, respectively. These yarn formulations were designed to achieve target fiber volume fractions in the consolidated laminates. The rCF/PA11 72/28 formulation targeted approximately 55 vol.% fiber, accounting for carbon-fiber losses during carding and additional polymer introduced through the wrapping filament and TFP stitching yarn. A higher-fiber-content rCF/PA11 80/20 variant targeted approximately 58 vol.% fiber to assess whether the increased fiber content could be consolidated with low porosity and to evaluate its effect on the mechanical response. The rCF/PA6 70/30 formulation was included as a reference material system using the same rCF and identical wrap-spinning parameters.

### 2.3. TFP Preforming, Consolidation, and Thermoforming

Flat coupon preforms and shaped demonstrator preforms were produced via TFP technology. The preforms were manufactured on industrial-grade embroidery machines. The rCF/PA11 yarn was placed along prescribed paths and secured using a PA11 stitching yarn on a water-soluble poly(vinyl alcohol) (PVA) backing, which provided dimensional stability during stitching while allowing removal prior to consolidation. The PVA backing was dissolved in water at 50 °C for 45 min, residual PVA was removed by rinsing at approximately 20 °C for 1–2 min, and the preforms were dried for 12 h at 65 °C. This step prevents residual PVA from interfering with PA11 melt consolidation or degrading the fiber–matrix interface. Symmetric stacking sequences were used where possible to reduce process-induced warpage during cooling.

Flat coupon panels and connecting-rod-shaped structural benchmarks were consolidated in a force-controlled matched tool. The applied force was 20 kN over an effective projected area of 7850 mm^2^, corresponding to 25.48 bar. No pressure was applied during the initial heating ramp. The nominal cycle comprised heating from ambient temperature to 220 °C over approximately 11 min, a 5 min hold at a measured return-flow temperature of 210–213 °C, and cooling from approximately 213 °C to 30 °C within 12 min under controlled force. The shaped shell demonstrator was preheated at 220 °C for approximately 240–300 s, transferred to the press within approximately 5 s, closed in 3 s, held for 55 s, and opened after 30 s. The mold temperatures were approximately 60–75 °C, and the nominal forming pressure was 100 bar.

**Figure 3:**
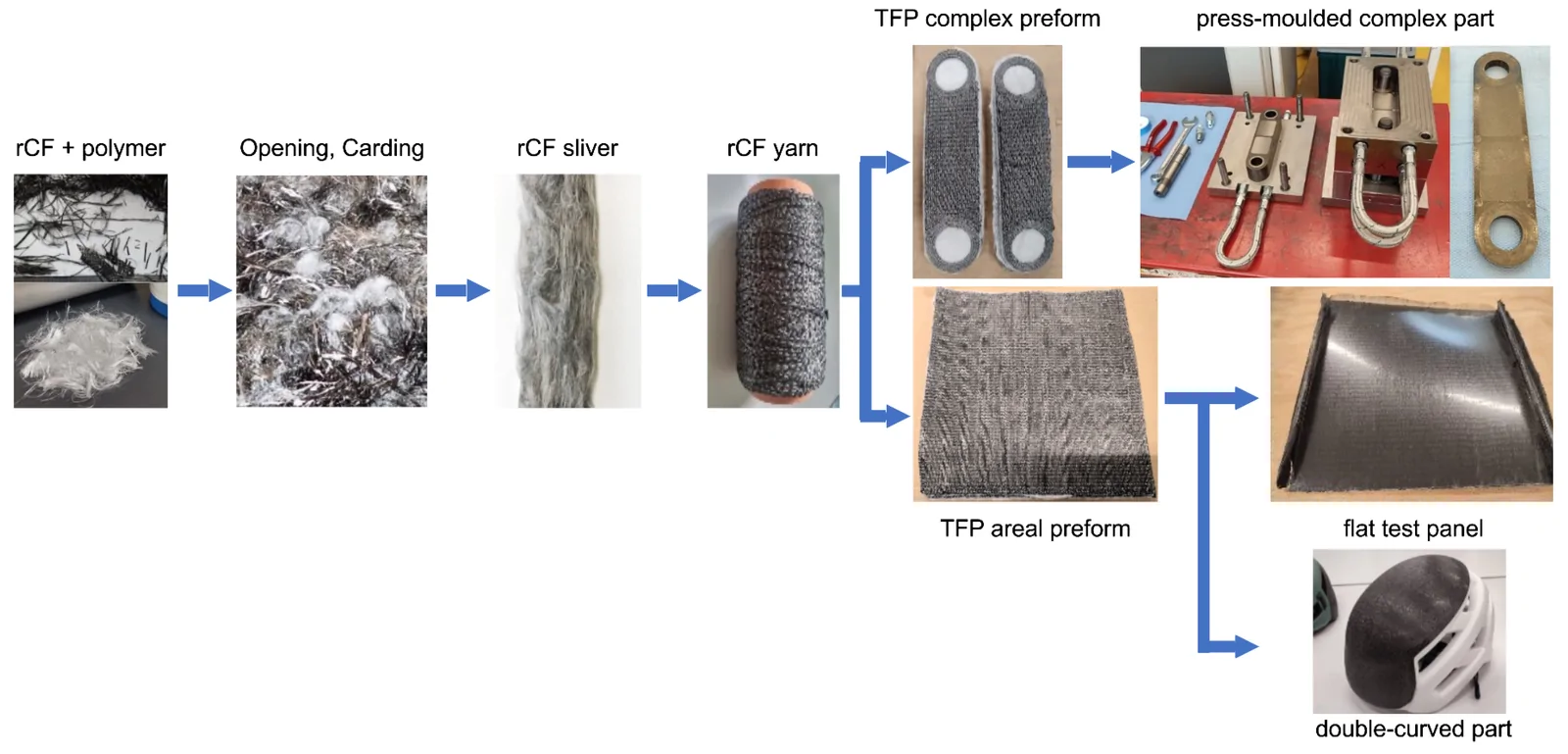
Overall rCF/PA11 processing route from staple fiber to consolidated flat laminates, shaped structural parts, and thermoformed double-curved shell demonstrators.

The processing routes from the rCF/PA11 wrap yarn to the consolidated components are summarized in Figure 3. Depending on the target geometry, TFP preforms were either directly consolidated into shaped structural parts, consolidated as flat laminates for coupon preparation, or first consolidated into flat organosheets and subsequently thermoformed into double-curved shell demonstrators.

### 2.4. Coupon Mechanical Characterization

Longitudinal and transverse tensile tests were performed on a ZwickRoell Z100 universal testing machine (ZwickRoell GmbH & Co. KG, Ulm, Germany). The procedure followed ASTM D3039 [27]. Tests were conducted at 23 °C and 2 mm/min. The grip pressure was 25 bar, the free specimen length between the grips was 156 mm, and strain was evaluated over a 50 mm gauge length. Specimens were fitted with 1 mm thick ±45° glass-woven tabs for a gentle load introduction. Multiple specimens were tested for each material condition, with the final valid specimen count depending on the exclusion of tests affected by grip failure or premature tab failure.

Additionally, in-plane shear response was determined for the rCF/PA11 72/28 material using a ±45° tensile configuration and evaluated using the stress–strain definitions of ISO 14129 [28]. The reported parameters were the in-plane shear modulus G_12_, maximum shear stress τ_12_^M^, and shear strain at maximum stress γ^M^.

### 2.5. Fiber Mass Content, Fiber Volume Content, and Microstructure

Fiber mass content was determined by selective dissolution of the polyamide matrix. Composite specimens were immersed in a 50/50 vol.% mixture of dichloromethane and formic acid and stirred magnetically until the polymeric constituents were dissolved. The remaining carbon fibers were recovered by vacuum filtration using a pre-weighed Whatman glass-microfiber filter and Büchner funnel, dried in a vacuum oven at 60 °C, and weighed. Fiber volume content was calculated from fiber mass content using the carbon-fiber and matrix densities:

(1) $$V_{f}=\frac{\frac{w_{f}}{\rho_{f}}}{\frac{w_{f}}{\rho_{f}}+\frac{\left(1-w_{f}\right)}{\rho_{m}}}$$

where *V_f_* is the fiber volume fraction, *w_f_* is the measured fiber mass fraction, *ρ_f_* is the carbon fiber density, and *ρ_m_* is the polymer density. Polished longitudinal sections were examined by optical microscopy to assess yarn-lane architecture, matrix distribution, local waviness, fiber-rich zones, and visible voids. Microscopy was used for qualitative microstructural assessment; no image-based void-content value was determined.

### 2.6. Structural Benchmark and Load-Introduction

**Figure 4:**
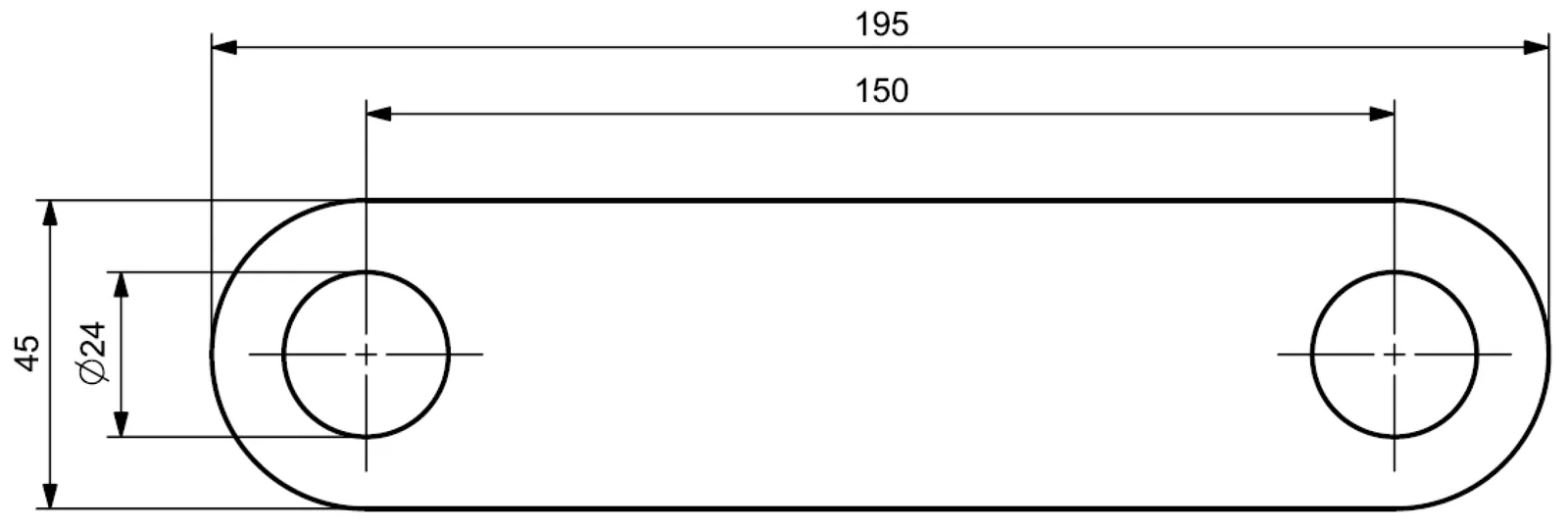
Schematic geometry of the conrod-shaped benchmark.

A conrod-shaped, two-hole benchmark geometry (Figure 4) was used to evaluate the material response beyond coupon-level testing in a load-bearing structural configuration (see also Figure 3). The specimen had an overall length of 195 mm, a hole-to-hole (gauge) distance of 150 mm, a nominal width of 45 mm, and two Ø24 mm end holes for pin loading. Specimens were loaded quasi-statically in the longitudinal direction through the end holes.

Virgin-CF/PA12 TFP preforms (thicknesses of 2.1 mm and 2.7 mm), virgin CF/epoxy RTM samples, and aluminum AW 6082 samples were included as references. Because the reference parts varied in thickness and mass, both absolute and normalized performance metrics were considered.

For each configuration, the maximum force (*F*_max_), sample mass (*m*), and thickness were recorded, and two derived metrics were evaluated. Nominal structural stress is

(2) $$\sigma_{\mathrm{nom}}=\frac{F_{\max}}{S_{0}},$$

where *S*_0_ is the nominal cross-sectional area of the gauge section (*S*_0_ = width × thickness = 45 mm × thickness), and the specific load capacity is

(3) $$\mathrm{Specific}\;\mathrm{load}\;\mathrm{capacity}=\frac{F_{\max}}{m},$$

where *m* is the mass of the individual sample. Because contact conditions, washer geometry, thickness, and local bearing stresses all influence *F*_max_, nominal stress is interpreted as a benchmark-specific performance metric rather than as an intrinsic material property.

### 2.7. Screening Life-Cycle Assessment

A screening life-cycle assessment (LCA) was performed for one connection-rod-shaped benchmark part. A screening-level approach was considered appropriate at this stage of development, since the goal was to obtain an early comparison between process concepts and identify relative hotspots that can guide subsequent material and process selection rather than to support a public comparative assertion between products. The supplied calculation separated contributions from carbon-fiber or rCF feedstock, matrix polymer, yarn manufacture, TFP preforming, compression molding, and RTM where applicable. Four routes were compared: rCF/PA11, vCF/PA12, rCF/PA6, and epoxy/virgin-CF. The indicator was reported as kilograms of CO_2_-equivalent per part. The calculation was used to identify relative hotspots and compare process concepts. The supplied documentation did not specify the background database, allocation method for rCF, electricity mix, cut-off rules, or uncertainty; consequently, the values are presented as a screening LCA rather than an ISO-conformant public comparative assertion. The final submission should document the goal, scope, functional unit, system boundary, inventory sources, and impact-assessment method in accordance with ISO 14040, ISO 14044, and ISO 14067 [29,30,31].

### 2.8. Comparison with the Published PA11/rCF Tape Architecture [25]

The yarn results at coupon scale were compared with the previously published rCF/PA11 tape data [25]. To reduce differences associated with fiber content, the primary comparison used the tape formulation containing a nominal fiber mass fraction of 70 wt.% rCF, resulting in 56.72 vol.% fiber, which lies between the yarn laminates at fiber volume fractions of 54.39 vol.% and 58.51 vol.%. The comparison included longitudinal and transverse tensile moduli and strengths and, where available, in-plane shear properties. Percentage differences were calculated relative to the reported tape values.

## 3. Results

### 3.1. Fiber Volume Content and Yarn-Lane Microstructure

Chemical dissolution resulted in average fiber mass contents of 67.58 wt.% for rCF/PA11 72/28, 71.13 wt.% for rCF/PA11 80/20, and 69.73 wt.% for rCF/PA6 70/30. The corresponding fiber volume contents were 54.39, 58.51, and 59.35 vol.%, respectively. For the rCF/PA11 formulations, the measured fiber mass fractions were lower than the nominal staple-fiber blend ratios. This difference is consistent with carbon-fiber losses during carding and the additional polymer introduced through the wrapping filament and TFP stitching yarn. In contrast, the measured fiber mass fraction of 69.73 wt.% for rCF/PA6 70/30 closely matched the nominal 70 wt.% rCF content.

**Figure 5:**
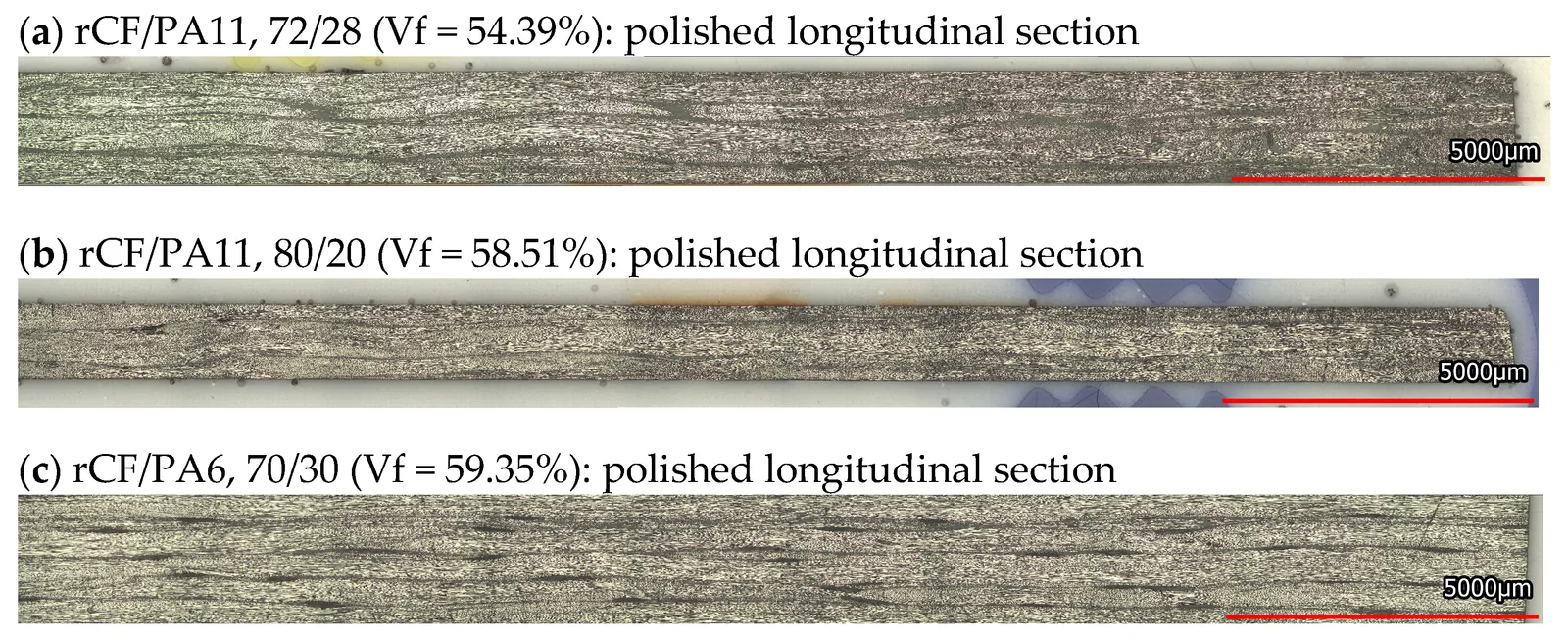
Microstructure of the yarn-derived laminates: representative polished longitudinal sections of (**a**) rCF/PA11 72/28, (**b**) rCF/PA11 80/20, and (**c**) rCF/PA6 70/30 laminates.

The longitudinal sections showed a dense, preferentially aligned architecture with visible yarn lanes and local bundle-scale waviness. The rCF/PA11 72/28 laminates (Figure 5a) exhibited a comparatively homogeneous microstructure, whereas the 80/20 material (Figure 5b) contained more pronounced matrix-poor lanes and local surface irregularities. The rCF/PA6 70/30 reference laminate (Figure 5c) showed a similarly aligned architecture but a visually different matrix distribution. The micrographs support the interpretation that the composite response is governed by two structural scales: alignment within the staple-fiber yarn core and interaction between adjacent yarn lanes after TFP and consolidation.

### 3.2. Coupon-Scale Mechanical Performance

**Figure 6:**
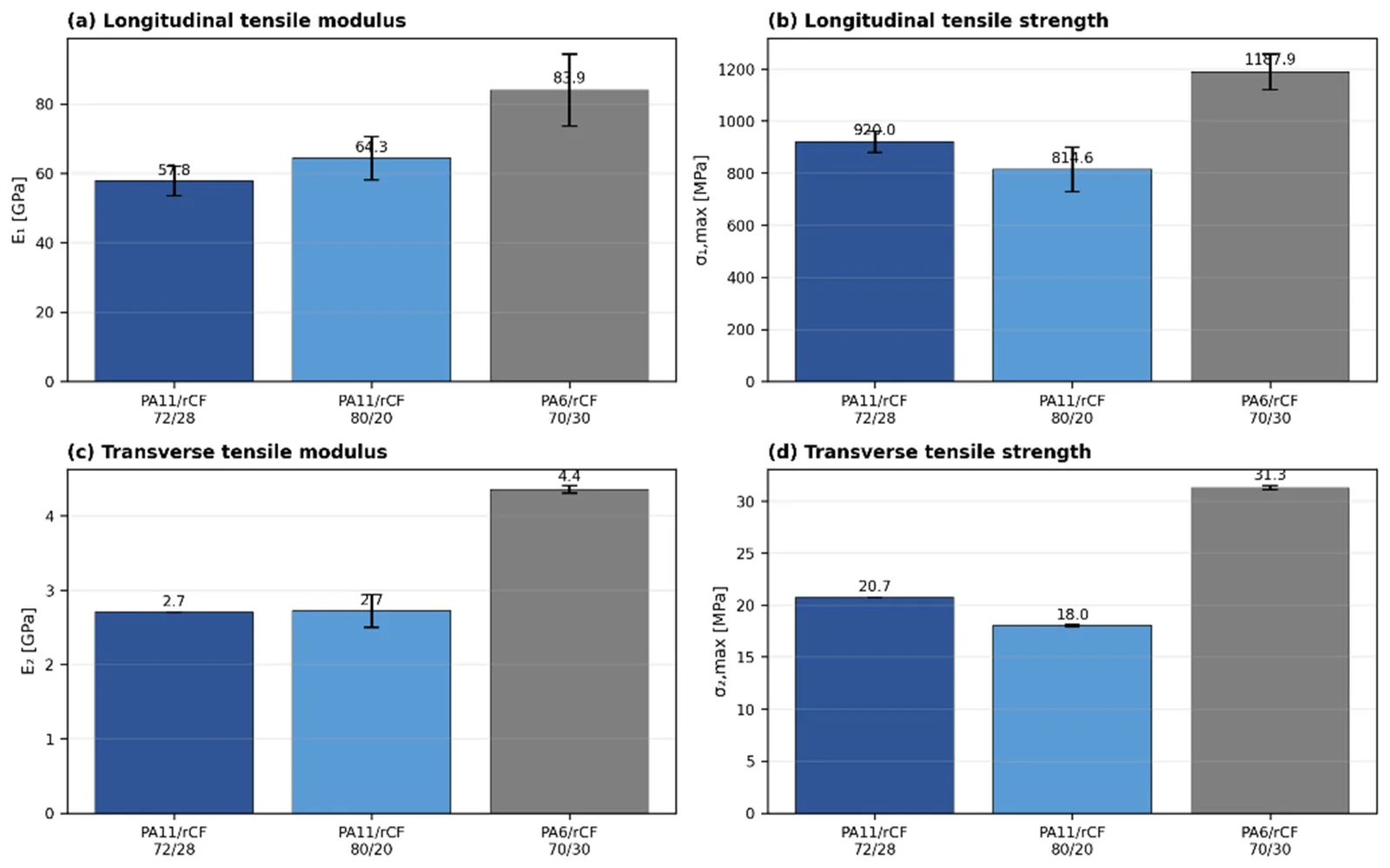
Coupon-scale tensile properties of the yarn-derived rCF/PA11 and rCF/PA6 laminates: (**a**) longitudinal tensile modulus; (**b**) longitudinal tensile strength; (**c**) transverse tensile modulus; and (**d**) transverse tensile strength.

The tensile results are summarized in Table 3 and Figure 6, and they are reported as mean values ± standard deviations.

**Figure 7:**
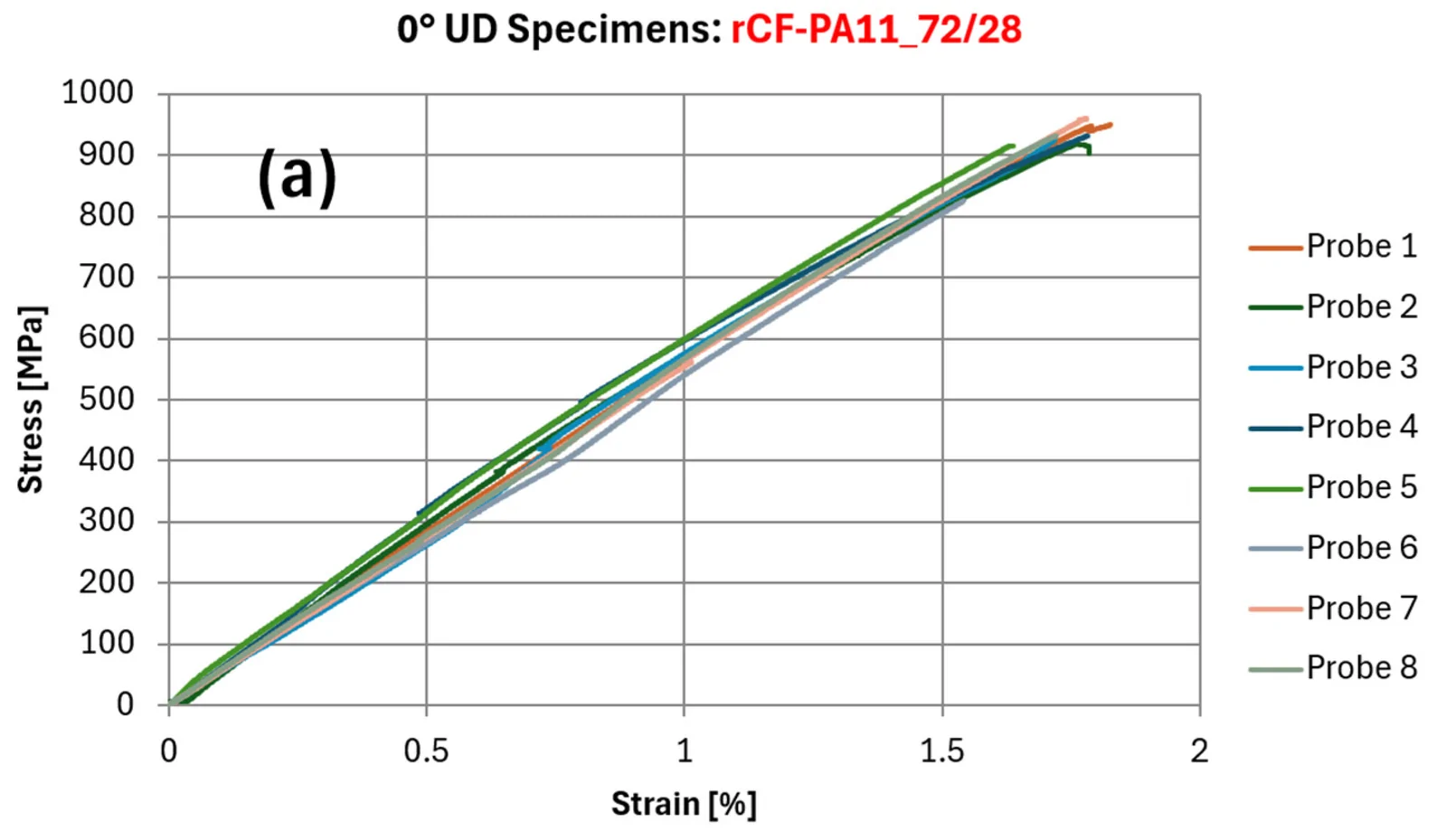

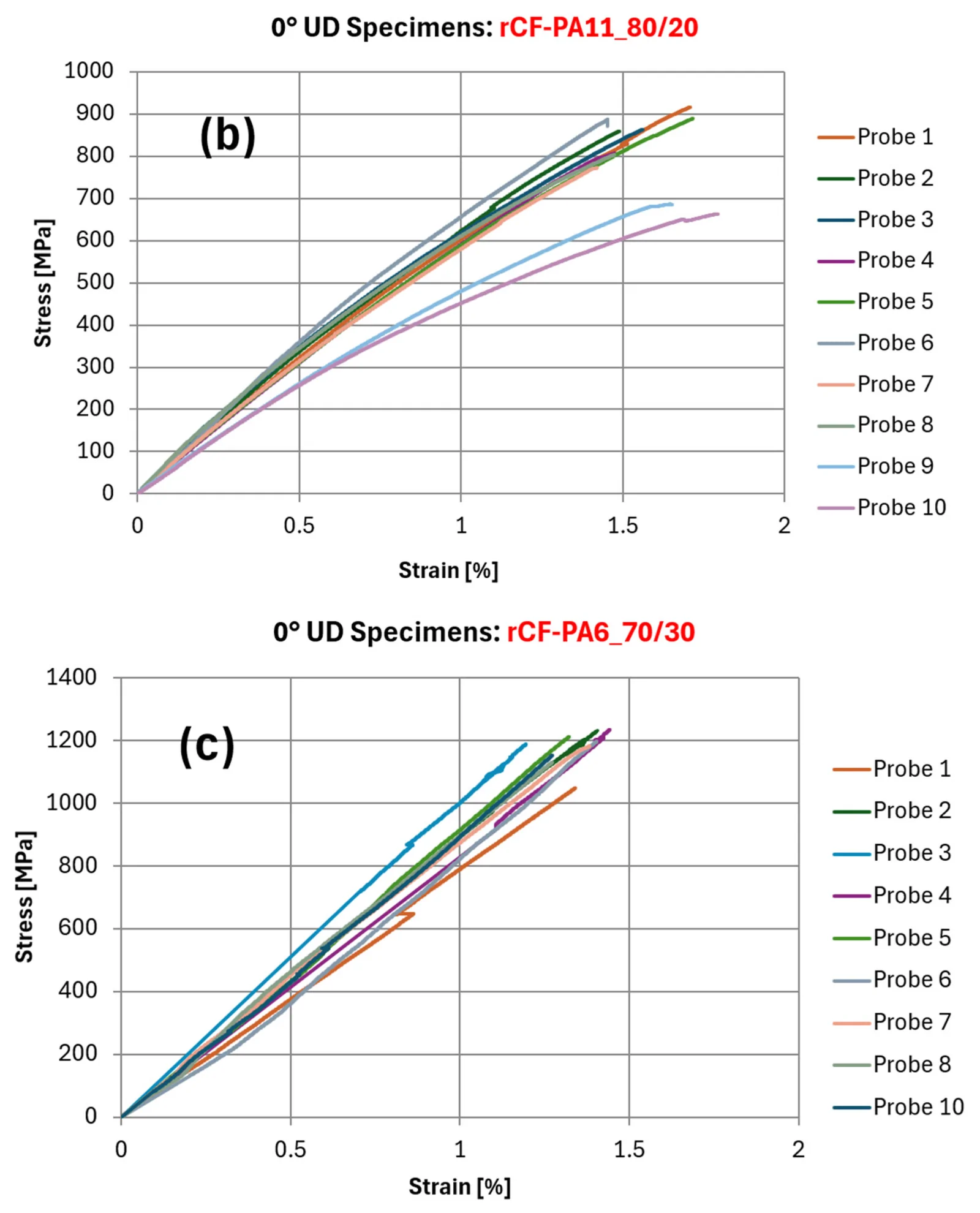
Stress–strain curves for 0° UD specimens: (**a**) PA11/rCF 72/28, (**b**) PA11/rCF 80/20, and (**c**) PA6/rCF 70/30 laminates.

The corresponding stress–strain curves for the 0° UD axial specimens are shown in Figure 7a–c. rCF/PA11 72/28 reached a longitudinal tensile modulus of 57.77 ± 4.23 GPa, a tensile strength of 920.0 ± 40.7 MPa, and an axial strain at break of 1.69 ± 0.10%. The higher-fiber-content rCF/PA11 80/20 laminate, with a measured fiber volume fraction of 58.51 vol.% compared with 54.39 vol.% for the 72/28 laminate, exhibited a higher longitudinal modulus of 64.31 ± 6.28 GPa but a lower mean tensile strength of 814.6 ± 85.6 MPa and a strain at break of 1.61 ± 0.24%. The higher stiffness but lower strength and larger scatter indicate that additional fiber content did not translate directly into improved defect tolerance. This increased scatter in the 80/20 group is driven largely by two specimens (Probes 9 and 10), which show a visibly lower stiffness above approximately 0.4% strain compared to the rest of the batch, likely reflecting local variability in fiber content or alignment. It is noted that probes 9 and 10 in Figure 7b were included in the overall dataset statistics as valid mechanical tests, with their earlier nonlinear stiffness degradation at higher strains reflecting localized microstructural variability and fiber alignment dispersion inherent to recycled staple fiber hybrid yarns rather than testing artifacts.

The rCF/PA6 70/30 reference exhibited the highest longitudinal tensile properties, with a modulus of 83.89 ± 10.36 GPa and a strength of 1187.9 ± 68.0 MPa. One specimen (Probe 9) was excluded from the analysis because its response was inconsistent with the remainder of the batch and was attributed to a testing artifact; the reported statistics therefore represent nine valid specimens. One possible contribution to the higher longitudinal properties is the greater stiffness of PA6 compared with PA11. In discontinuous-fiber composites, the matrix contributes to stress transfer between adjacent fibers; within a shear-lag framework, a stiffer matrix can promote more effective axial load transfer and thereby contribute to higher laminate stiffness and strength.

Much fewer specimens were tested in the transverse direction, and the corresponding stress–strain curves are therefore not presented here given the limited sample size. The transverse results are thus exploratory because replication was limited. The 72/28 transverse values represent one valid specimen, for which no standard deviation or confidence interval can be estimated. For rCF/PA11F 80/20 and rCF/PA6 70/30 transverse properties, the reported scatter in Table 3 describes the tested specimens and does not necessarily establish batch-to-batch reproducibility.

Transverse tensile properties were substantially lower for all variants than in the longitudinal direction. The rCF/PA11 80/20 laminate reached a transverse modulus of 2.72 ± 0.22 GPa and strength of 18.0 ± 0.1 MPa, while the rCF/PA6 70/30 reference material reached 4.36 ± 0.05 GPa and 31.3 ± 0.2 MPa. For the rCF/PA11 72/28 laminate, the single valid transverse specimen yielded a modulus of 2.70 GPa and a strength of 20.7 MPa. The pronounced difference between longitudinal and transverse properties is consistent with the strongly oriented reinforcement structure observed in the longitudinal micrographs.

**Figure 8:**
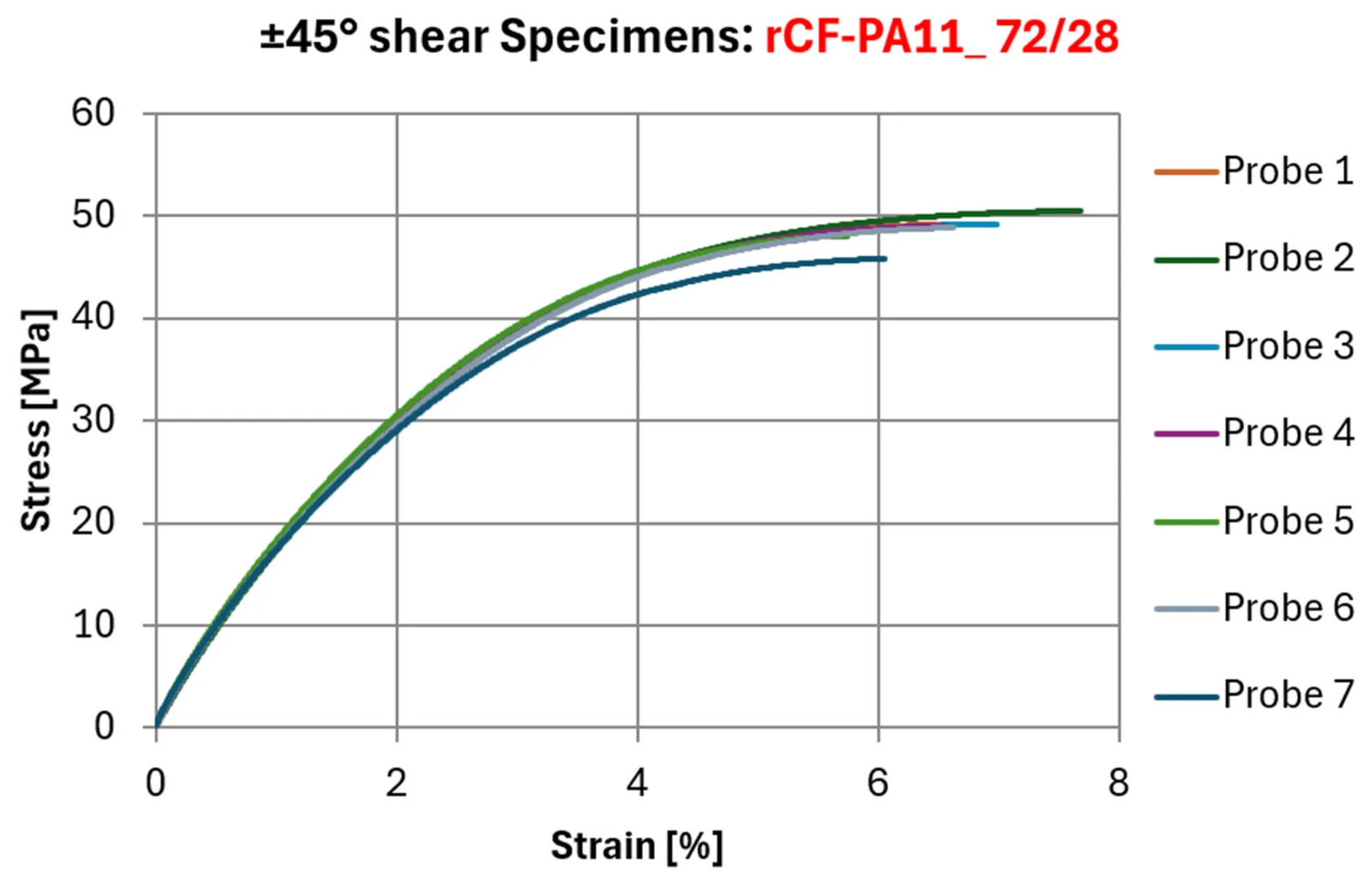
In-plane shear stress–strain curves obtained from ±45° tensile specimens of rCF/PA11 72/28.

The ±45° shear response was characterized only for the rCF/PA11 72/28 material system, shown in Figure 8 with *n* = 7 valid specimens. The resulting in-plane shear modulus G_12_ was 1.874 ± 0.033 GPa, the maximum shear stress τ_12_^M^ was 48.7 ± 1.4 MPa, and the shear strain at maximum stress was γ^M^ = 6.59 ± 0.63%.

The shear response was nonlinear and approached a plateau near the maximum stress, indicating progressive matrix and interface deformation rather than an abrupt fiber-dominated failure. As shown in Figure 8, the individual specimen curves closely overlapped up to approximately 3% strain, with only Probe 7 deviating to a lower plateau stress at larger strains, reflecting the generally low scatter observed across the yarn-based specimens.

Overall, the longitudinal and shear results show comparatively tight scatter across replicate specimens, with the exception of two specimens within the 80/20 longitudinal group, suggesting good process consistency in the yarn-based consolidation route. Achieving consistent mechanical properties in addition to high absolute performance is an important requirement for textile-processed aligned rCF composites, as local variability in yarn and laminate structure may contribute to specimen-to-specimen scatter. The consistently low scatter observed here across the longitudinal and shear datasets indicates that the processing route achieves a good degree of uniformity, supporting its accuracy and reproducibility even when using recycled fiber input.

### 3.3. Comparison with Previously Published rCF/PA11 Tape Results

**Figure 9:**
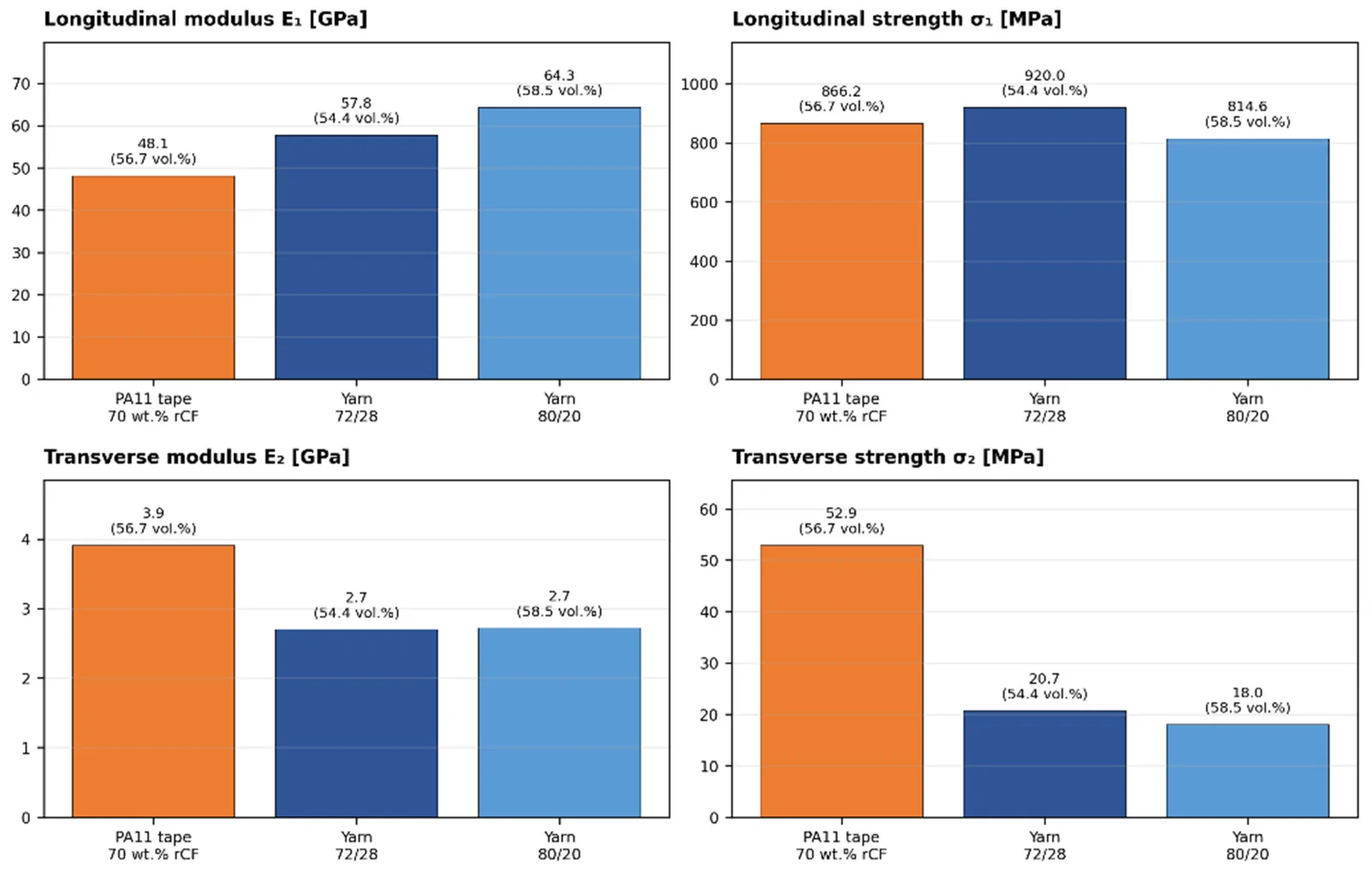
Comparison of the published rCF/PA11 tape material [25] with the rCF/PA11 yarn laminates. Values in parentheses give the fiber volume content determined by chemical dissolution.

Figure 9 and Table 4 compare the yarn laminates with the published rCF/PA11 tape material at similar fiber volume contents [25]. rCF/PA11 72/28 yarn had a fiber volume content of 2.33 percentage points below the tape yet achieved a 20.0% higher longitudinal modulus and a 6.2% higher longitudinal strength. The rCF/PA11 80/20 yarn, with 1.79 percentage points higher fiber volume content than the tape, achieved 33.6% higher longitudinal modulus but 6.0% lower strength. These differences indicate that the present yarn-based laminates achieved longitudinal properties within or above the range reported for the earlier tape system. However, the observed differences cannot be attributed to intermediate architecture alone because the studies differed in carding equipment, process maturity, staple-fiber characteristics, and subsequent processing conditions. The comparison should therefore be interpreted as literature context rather than as a controlled yarn-versus-tape assessment.

The matrix-dominated properties showed the opposite trend. Relative to the reported tape values, the 72/28 yarn exhibited a 31.0% lower transverse modulus, a 60.9% lower transverse strength, a 47.4% lower shear modulus, and a 58.2% lower shear strength. The 80/20 yarn showed a similar reduction in transverse properties. This reduction is consistent with differences in fiber-orientation distribution and matrix continuity between the two material systems. The reported tape material may exhibit greater off-axis fiber orientation [25] and a more continuous matrix phase, which could contribute to improved transverse and shear load transfer while potentially reducing the degree of axial reinforcement efficiency. The chosen wrap yarn structure promotes a high degree of reinforcement alignment along the yarn direction, which may contribute to the comparatively high longitudinal properties, while the discrete yarn-lane structure may be less favorable for transverse and shear load transfer.

### 3.4. Conrod-Shaped Structural Benchmark

**Figure 10:**
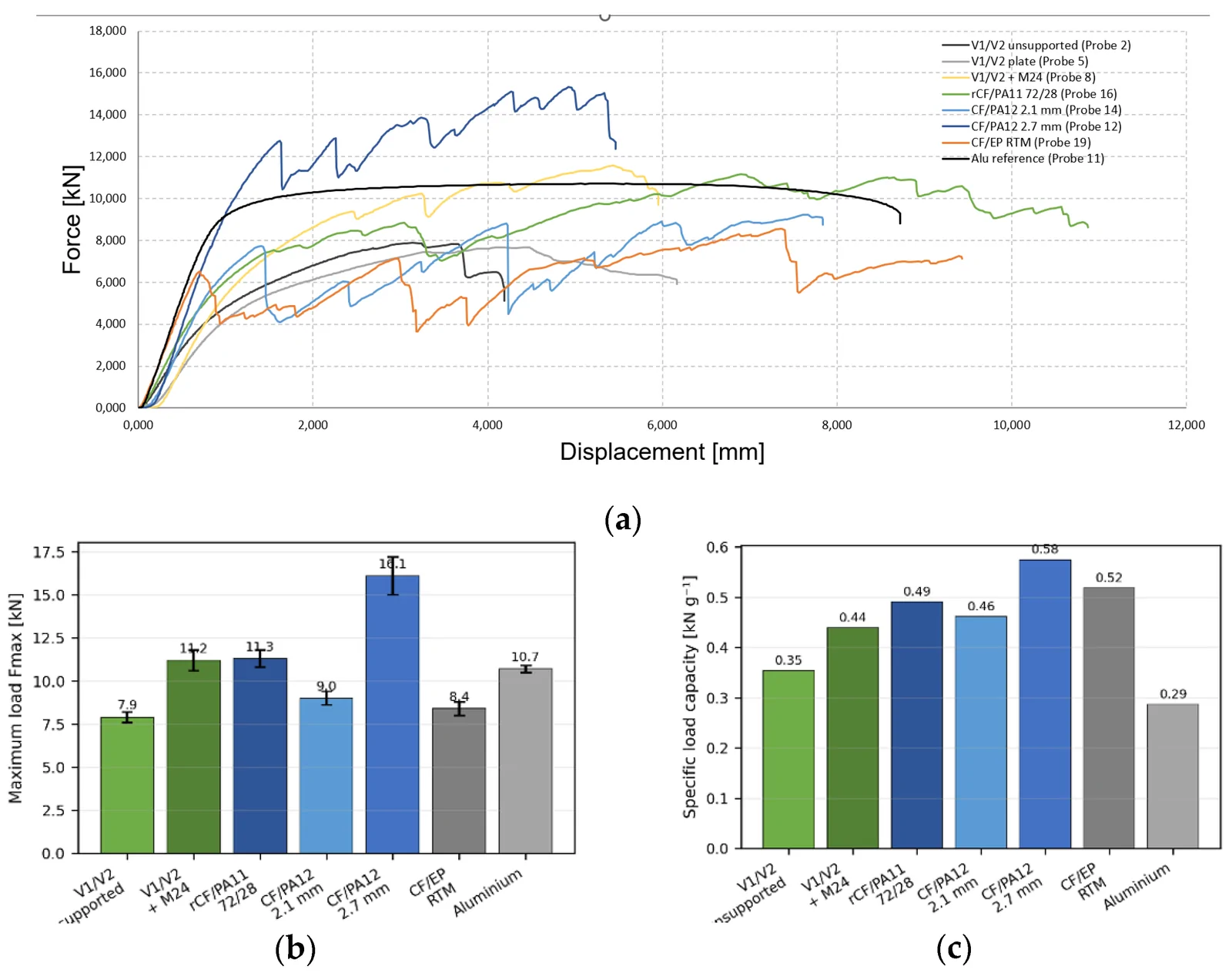
(**a**) Representative force-displacement responses; (**b**) maximum load; (**c**) specific load capacity.

The structural benchmark results (see Section 2.6) are listed in Table 5 and shown in Figure 10. In addition to the rCF/PA11 72/28 configuration selected for the final benchmark assessment, an rCF/PA11 70/30 variant was tested in several load-introduction configurations during the development of the benchmark setup. The parameter *n* in Table 5 denotes the number of tested specimens. The highest absolute load was obtained for the vCF/PA12 reference (with 2.7 mm thickness) at 16.1 ± 1.1 kN. The best rCF result was the supported rCF/PA11 72/28 benchmark specimen at 11.3 ± 0.5 kN, slightly exceeding the aluminum reference at 10.7 ± 0.2 kN. Because the rCF/PA11 specimen weighed 23.0 ± 1.0 g compared with 37.3 ± 0.6 g for aluminum, its specific load capacity was 0.491 kN g^−1^ versus 0.287 kN g^−1^ for aluminum.

**Figure 11:**
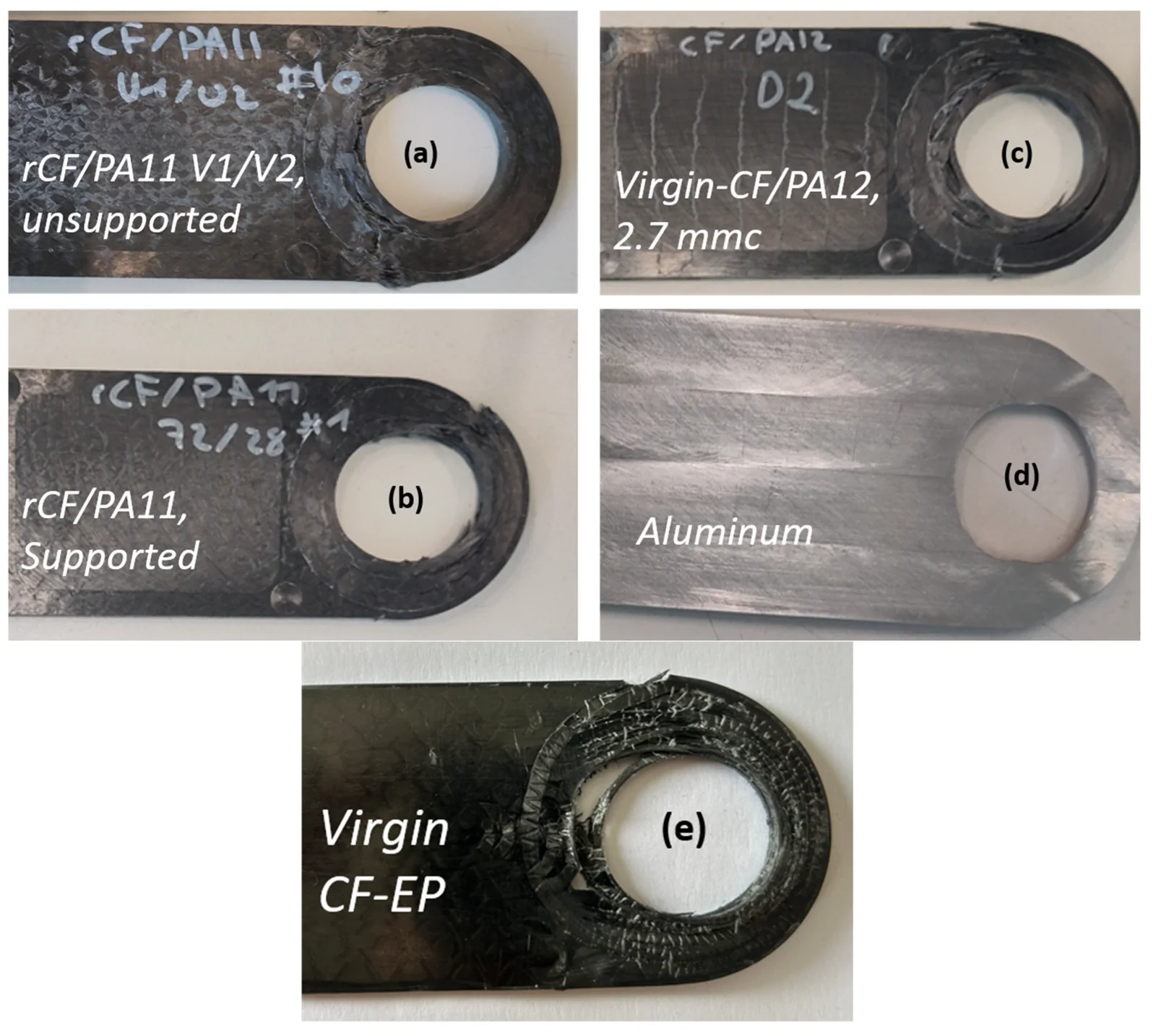
Representative post-test conrod-shaped specimens showing hole-region failure: (**a**) rCF/PA11 70/30, unsupported; (**b**) rCF/PA11 72/28, supported; (**c**) vCF/PA12; (**d**) Aluminum; (**e**) vCF/EP. All specimens show damage initiating at the loaded hole and propagating toward the specimen edge.

Figure 11 shows representative post-test specimens for the investigated benchmark configurations. All materials failed due to damage initiated at the loaded hole, which propagated as splitting toward the free edge, consistent with a combined bearing/net-tension failure mode at the load-introduction point. The extent of visible cracking and delamination varied between configurations: The unsupported rCF/PA11 specimen showed pronounced splitting extending well beyond the hole region, whereas the supported rCF/PA11 72/28 and virgin-CF/PA12 specimens exhibited more localized damage confined closer to the hole edge. These observations indicate that the load-introduction conditions, in addition to laminate properties, influence the observed damage progression and maximum load.

The load-introduction study showed a pronounced influence of the local support configuration. The original support plate alone did not improve rCF/PA11 70/30’s maximum force, but the combination of the plate and M24 washers increased Fmax from 7.9 to 11.2 kN, a gain of approximately 41%. Force–displacement curves showed stepwise drops associated with progressive local damage. Post-test inspection identified damage around the loading holes and longitudinal splitting. Consequently, load introduction, bearing area, and washer stiffness should be standardized before the part can be used for a material-ranking test. Nevertheless, the supported 72/28 result demonstrates a favorable combination of load and mass: relative to aluminum, Fmax increased by 5.6%, mass decreased by 38%, and specific load capacity increased by 71%.

### 3.5. Double-Curved Thermoformed Demonstrator

**Figure 12:**
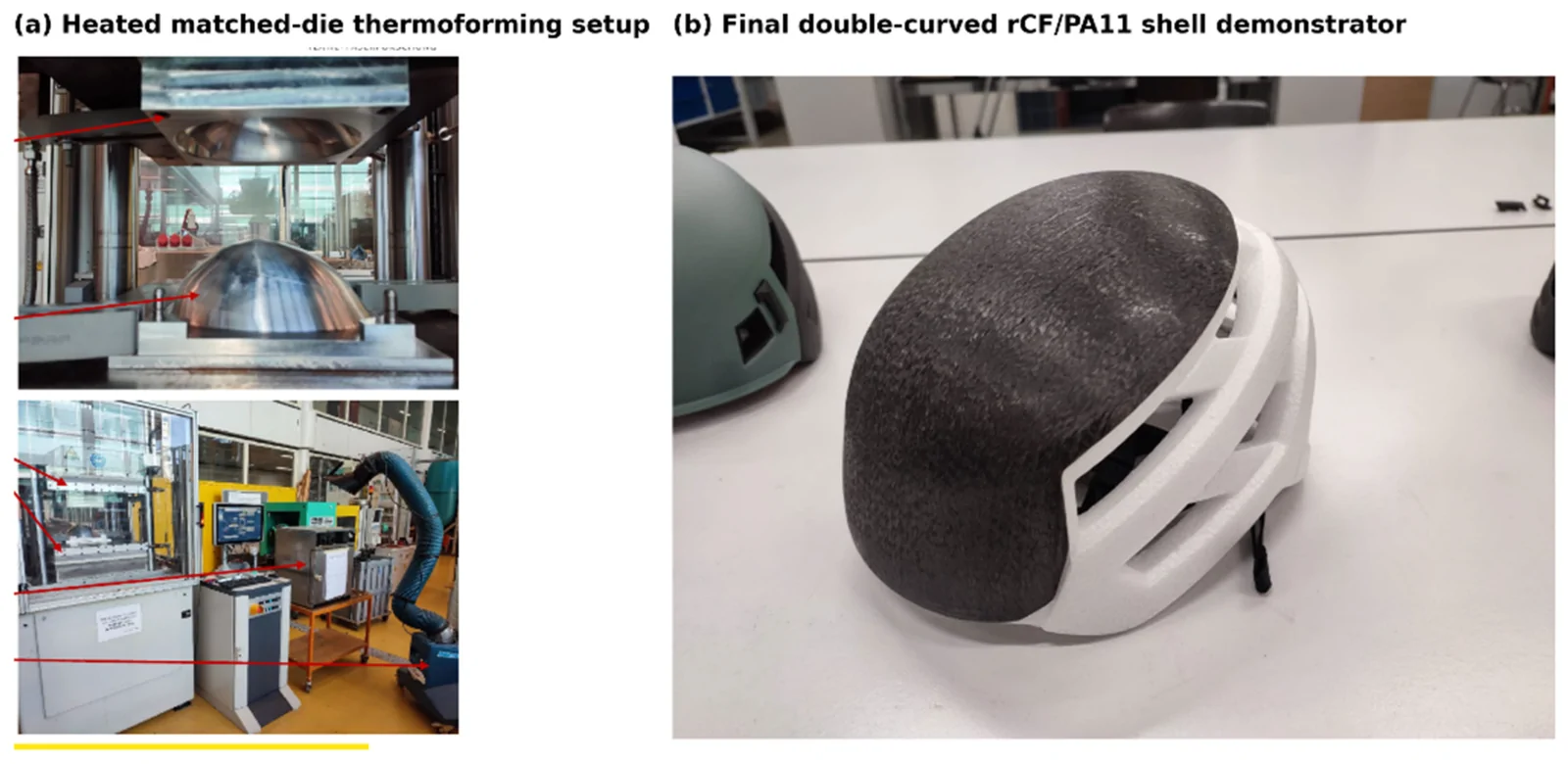
Double-curved demonstrator manufacture: (**a**) heated matched-die thermoforming setup (The red arrows indicate the key components and processing areas of the thermoforming equipment) and (**b**) final yarn-based rCF/PA11 shell demonstrator.

Three double-curved shell demonstrators (Figure 12) were successfully thermoformed from the yarn-based TFP organosheets. The relatively bulky wrap-yarn structure may facilitate local rearrangement and nesting of adjacent yarn lanes during forming, thereby helping the preform accommodate the imposed three-dimensional geometry. The matched-die process reproduced the global shell geometry without gross tearing or loss of structural integrity. Visible surface and alignment defects remained, particularly in regions requiring large in-plane shear and material draw-in. These defects indicate that TFP path design, preform boundary shape, stitching density, and local yarn compaction require further optimization for complex curvatures.

A fourth preform was not processed because broken needle elements remained entangled in the TFP preform and posed a tool-damage risk. This observation highlights a manufacturing-quality requirement that is not captured by coupon-level testing: automated inspection and removal of stitching or backing artifacts are necessary before high-pressure forming. Overall, the successful thermoforming of three demonstrators validated the formability potential of the yarn architecture while also identifying preform quality assurance as a critical scale-up topic.

### 3.6. Screening Life-Cycle Assessment Results

**Figure 13:**
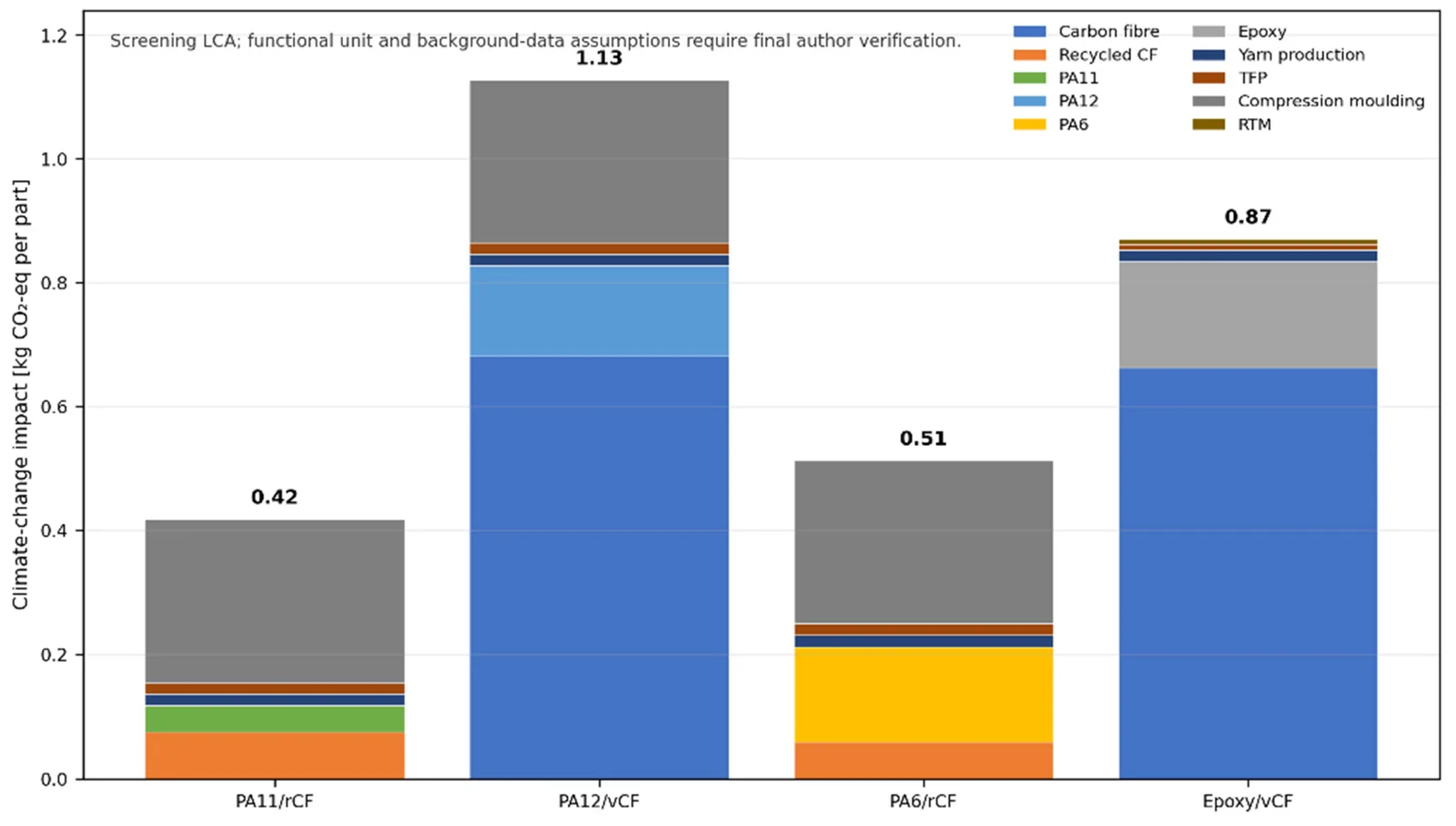
Screening climate-change impact of four benchmark-part manufacturing routes, separated into material and process contributions.

The screening LCA results for the climate-change indicator are summarized in Table 6 and Figure 13. The rCF/PA11 route had the lowest calculated impact at 0.417 kg CO_2_-eq per benchmark part, followed by rCF/PA6 at 0.513 kg CO_2_-eq. The virgin-CF/epoxy and virgin-CF/PA12 routes resulted in 0.870 and 1.128 kg CO_2_-eq per benchmark part, respectively. Under the supplied assumptions, the calculated climate-change impact of the rCF/PA11 route was 63.0% lower than that of virgin-CF/PA12, 52.1% lower than that of virgin-CF/epoxy, and 18.6% lower than that of rCF/PA6.

Compression molding was the largest single contribution in the rCF/PA11 route, accounting for approximately 63% of its total calculated climate-change impact. In the virgin-CF routes, carbon-fiber production dominated. The results indicate that the lower calculated impact associated with the rCF routes was maintained despite the additional yarn-manufacturing and TFP steps. For the rCF/PA11 route, compression-molding energy therefore represents the dominant remaining process contribution and an important target for further process optimization. This is consistent with LCA studies of aligned discontinuous-fiber remanufacturing, which identify process electricity as an important hotspot [32].

The present environmental screening is conducted specifically for the physical mass, thickness, and geometric configuration of the tested conrod demonstrator parts. Because the compared manufacturing routes involve variations in component mass, thickness, and static load-bearing performance, these results reflect configuration-specific hotspot metrics rather than an ISO-compliant functional unit comparison across standardized structural components. Consequently, these findings serve to highlight processing energy and emissions hotspots within the yarn-based manufacturing chain rather than establishing a generalized, material-level environmental ranking.

## 4. Discussion

### 4.1. Fiber Volume Content and the Axial Stiffness/Strength Trade-Off

The yarn route achieved fiber volume fractions of 54.4 vol.% for rCF/PA11 72/28 and 58.5 vol.% for rCF/PA11 80/20, closely matching the respective target values. These values are higher than those typically achieved in short-fiber compounds and within the range commonly reported for continuous-fiber thermoplastic laminates. The longitudinal moduli of 57.8–64.3 GPa for yarn-based rCF/PA11 and 83.9 GPa for rCF/PA6 indicate effective utilization of carbon-fiber stiffness along the yarn direction. These values are also substantially higher than the longitudinal moduli of approximately 32–48 GPa reported in the earlier rCF/PA11 tape study at lower and similar fiber contents [25].

However, the rCF/PA11 80/20 material illustrates that increasing fiber content does not necessarily translate into higher measured tensile strength. Although its fiber volume content and longitudinal modulus exceeded those of the 72/28 material, its tensile strength was approximately 11% lower, with a standard deviation more than twice as high. This behavior may reflect greater sensitivity at the higher fiber volume fraction to local defects, fiber waviness, inter-yarn regions, and reduced matrix availability. Thus, the higher fiber volume fraction was associated with increased longitudinal stiffness but did not translate into a corresponding increase in tensile strength under the processing conditions investigated. The 72/28 material was therefore selected for the demonstrator because it provided the most favorable combination of strength, stiffness, and processability, and lower tensile-strength scatter than the 80/20 formulation.

It should also be noted that while high longitudinal moduli and high consolidated fiber volume fractions provide clear quantitative proof of preferential axial fiber alignment [12], a full 3D Fiber Orientation Distribution (FOD) tensor was not explicitly measured. Quantification via 2D optical cross-sectional ellipse analysis remains limited by cut-plane bias and small sampling volumes. For future work, particularly when developing high-fidelity micromechanical constitutive models or predicting anisotropic strength boundaries, full 3D X-ray micro-CT volumetric imaging or automated digital image orientation mapping [7] should be implemented to extract exact FOD tensors across the bulk preform architecture.

### 4.2. Comparison of Longitudinal and Matrix-Dominated Performance in Yarn- and Tape-Based rCF/PA11 Laminates

The comparison with the previously published rCF/PA11 tape study provides useful context for interpreting the balance between longitudinal and matrix-dominated properties. At similar FVC, the 72/28 yarn laminate exceeded the tape in longitudinal modulus and strength. The yarn-forming step concentrates the aligned staple fibers into a compact, approximately circular core, which may promote a high degree of reinforcement alignment along the yarn axis, while the helically wrapped filament stabilizes them during handling and TFP. Hasan et al. [15] further showed that yarn structure can influence the internal fiber-orientation distribution and local composite microstructure. In their comparison of wrap-spun and friction-spun rCF yarns, the wrap-spun architecture exhibited a broader fiber-orientation distribution and a larger fraction of fibers deviating from the yarn axis in the outer regions. This behavior was associated with the greater hairiness of the wrap-spun yarns, whereas the more compact friction-spun yarns showed larger regions of well-oriented fibers. Hasan et al. further observed more resin-rich regions in the wrap-spun composites, which they related to the more open yarn structure and greater resin infiltration. Thus, architecture-induced differences are not restricted to the comparison between tape- and yarn-based intermediates but can also be substantial between different yarn architectures themselves. The previously reported tape material may exhibit a broader in-plane fiber-orientation distribution and a more spatially continuous reinforcement arrangement. Such differences may contribute to the comparatively high longitudinal properties of the present yarn laminates.

In contrast to the higher longitudinal properties of the yarn laminate, its matrix-dominated transverse tensile and in-plane shear properties were lower than those reported for the tape laminate. Compared with the tape laminate, the yarn laminate contains discrete reinforcement lanes and yarn-to-yarn interfaces that are expected to influence the transverse load transfer and shear deformation. A high degree of axial fiber alignment may reduce the fraction of reinforcement contributing to off-axis loading, while the discrete yarn-lane structure may promote spatially nonuniform fiber and matrix distributions after consolidation. Numerically, the transverse tensile and in-plane shear strengths of the 72/28 yarn laminate were 61% and 58% lower, respectively, than those of the tape laminate. These differences are consistent with previous studies showing that yarn structure and processing parameters influence fiber orientation and local matrix distribution [12,13,14]. Accordingly, the comparison with the previously published tape study suggests a different balance between longitudinal and matrix-dominated properties for the present yarn-based laminates, but this should not be interpreted as a controlled ranking of yarn and tape architectures. The yarn-based route nevertheless offers processing advantages for continuous TFP placement, near-net-shape preforming, reinforcement-path control, and subsequent three-dimensional forming.

### 4.3. Structural Demonstrator: Material Performance Versus Load Introduction

The conrod-shaped benchmark showed that a yarn-based rCF/PA11 achieved an absolute maximum load comparable to that of aluminum at substantially lower specimen mass. The 72/28 part achieved 11.3 kN and a 71% higher load-to-mass ratio than aluminum. This benchmark extends the assessment beyond coupon-level material properties by incorporating holes, curved end regions, TFP path transitions, and pin-based load introduction. The result also compares favorably with the thinner virgin-CF/PA12 and virgin-CF/epoxy references on a mass-normalized basis.

At the same time, the load-introduction study demonstrates that the result is strongly governed by local support and bearing conditions. Increasing bearing area raised rCF/PA11 70/30 Fmax by 41%, without changing the base laminate. Progressive force drops and longitudinal splitting indicate mixed bearing, delamination, splitting, and net-section failure. A final design comparison therefore requires matched thickness, a fixed washer/support stack, documented failure modes, and ideally a local finite-element contact analysis. The present benchmark should be interpreted as a proof of structural potential and load-introduction sensitivity, not as a standard material test.

It is noted that the structural assessment of the TFP conrod demonstrator was strictly limited to quasi-static pin loading. No cyclic fatigue life, damage accumulation under dynamic fatigue, or endurance limits were evaluated. While the present results confirm baseline quasi-static load-bearing capability and tailored load-path execution, full suitability for repeated cyclic service loading requires a dedicated, multi-parameter fatigue campaign investigating various stress ratios (R), frequencies, and stress amplitudes. Comprehensive fatigue and impact testing of these rCF/PA11 architectures are explicitly recommended as vital directions for future work.

### 4.4. Three-Dimensional Forming Capability and Manufacturing Robustness

The successful production of three double-curved shells supports the potential of the yarn-based route to offer advantages over flat tape intermediates for complex three-dimensional thermoforming. Bulky yarn lanes may facilitate local rearrangement, nesting, and changes in yarn-lane spacing during heating and forming. This behavior may improve drape and pressure equalization but can also create visible alignment defects. The problem is therefore not simply whether the material can be formed, but whether the fiber paths remain within structural tolerances after forming. Deep-drawing studies of rCF/PA6 hybrid-yarn organosheets reported similar benefits from staple-fiber mobility and yarn architecture [19,20]. Future work should therefore combine TFP path compensation, local blank-holder control, forming simulation, and post-form fiber-orientation mapping.

The failed fourth preform also highlights the importance of manufacturing robustness. For serial production of TFP preforms, the process parameters must be tailored to the material to ensure process stability and minimize defects. Quality-control requirements have to be defined accordingly.

It is noted that the thermoformed shells successfully reproduced the complex double-curved 3D geometry without gross macro-tearing or surface consolidation failure. Local surface irregularities and yarn path deviations were visually observable along sharp radii; however, these localized fiber orientation angles and affected areas were not quantitatively mapped through the thickness. Consequently, these photographs demonstrate macro-scale geometric forming feasibility and drapeability rather than quantified internal fiber alignment or retained mechanical properties.

### 4.5. Environmental Interpretation and Limitations

The screening LCA indicates a substantially lower climate-change impact for the rCF/PA11 route under the applied assumptions. The calculated 63% reduction relative to the vCF/PA12 route is primarily attributable to the substitution of virgin carbon fiber by rCF, whereas the 19% reduction relative to rCF/PA6 reflects the lower matrix-related contribution assigned to PA11 in the supplied inventory. The rCF/PA11 route also showed a lower calculated climate-change impact than the vCF/epoxy route despite the energy demand for compression molding. These trends are consistent with established studies showing that rCF recovery and reuse can reduce energy demand and greenhouse gas emissions compared with virgin-CF production.

The numerical LCA results must nevertheless be treated as provisional. The source data did not define the rCF allocation rule, geographic electricity mix, database versions, transport, scrap rates, tool amortization, or end-of-life scenario. The functional unit was defined as one benchmark part; however, the compared parts were not fully equivalent in geometry or mechanical performance. The present results are valuable for hotspot identification: Within the rCF/PA11 route, press energy dominates the rCF/PA11 impact, so shorter cycles, lower thermal mass tooling, heat recovery, and renewable electricity are likely to offer the greatest improvements.

Additional limitations include the small transverse and structural-coupon sample sizes; the absence of quantitative void-content data; incomplete documentation of rCF provenance; and the lack of fatigue, impact, environmental aging, and repeated forming data. The comparison with the previously reported rCF/PA11 tape system provides useful context but does not constitute a controlled single-variable experiment because the two studies differed in carding equipment, process maturity, staple-fiber characteristics, and subsequent processing conditions.

## 5. Conclusions

This study established a yarn-based route for converting recovered carbon staple fiber and bio-based PA11 into aligned thermoplastic laminates and shaped demonstrators. The principal conclusions are as follows:

1. The wrap-yarn route achieved high fiber contents. Chemical dissolution gave 54.39 vol.% for rCF/PA11 72/28 and 58.51 vol.% for rCF/PA11 80/20.
2. rCF/PA11 72/28 achieved 57.77 ± 4.23 GPa longitudinal modulus and 920.0 ± 40.7 MPa strength. Increasing fiber content to the 80/20 formulation raised the modulus to 64.31 ± 6.28 GPa but reduced strength to 814.6 ± 85.6 MPa, suggesting increased defect sensitivity at a lower matrix content.
3. At comparable fiber volume content, the 72/28 yarn exceeded the published rCF/PA11 tape by approximately 20% in longitudinal modulus and 6% in longitudinal strength. In contrast, transverse and shear properties were markedly lower, confirming an architectural trade-off between efficient yarn-core reinforcement and inter-yarn matrix continuity. However, a direct attribution of these differences to intermediate architecture is not possible because the studies differed in carding equipment and process conditions, as well as in staple-fiber morphology and crimp.
4. The supported bone-shaped rCF/PA11 72/28 benchmark sustained 11.3 ± 0.5 kN at 23.0 ± 1.0 g. Relative to aluminum, it delivered 5.6% higher maximum load, 38% lower mass, and 71% higher specific load capacity. Load introduction remained a dominant design variable.
5. Three double-curved shell demonstrators were successfully thermoformed. The screening LCA estimated a global warming potential of 0.42 kg CO_2_-eq for the rCF/PA11 benchmark part, approximately 63% below vCF/PA12 and 52% below vCF/epoxy under the supplied assumptions. Compression-molding energy represented the dominant remaining contribution. The screening LCA indicates energy savings during fiber recovery and hybrid yarn spinning. However, because functional unit equivalency depends on part-specific mass, thickness, and performance requirements, these environmental benefits are configuration-specific and characterize the studied processing hotspot rather than a standardized component-wide LCA.
6. The results support manufacturing feasibility and preliminary quasi-static performance. Future work with further replications and the presence of quantitative fiber-length, orientation, and void distributions will support broader conclusions.

The yarn architecture therefore complements rather than replaces the rCF/PA11 tape architecture. Yarn is particularly attractive for TFP, load-path steering, and complex preforming.

## Figures and Tables

**Table 1:** Principal roller-carding parameters used for the rCF/polyamide staple-fiber blends.

Parameter	Value
Pre-opening passes	2
Roller-carding machine working width	1000 mm
Feeding grammage	250 g/m
Feeding belt speed	0.061 m/min
Nominal feed mass flow	15.25 g/min
Number of worker–stripper pairs	5
Main cylinder rotational speed	193 rpm
Licker-in: main cylinder speed ratio	1:39
Worker: stripper speed ratio	1:45
Doffer: main cylinder speed ratio	1:67
Target sliver linear density	2.0 ktex

**Table 2:** Principal wrap-spinning parameters used for the rCF/polyamide hybrid yarns.

Parameter	Value
Input sliver linear density	Approx. 2 ktex
Number of slivers fed	2
Total draft	3.5–6.8
Yarn delivery speed	10 m/min
Wrap density	160 wraps/m
Wrapping direction	Z
Target yarn linear density	800 tex

**Table 3:** Tensile properties of yarn-derived rCF/polyamide laminates.

Material	Orientation	*n* (Number of Valid Tests)	Modulus [GPa]	Strength [MPa]	Strain at Break [%]
rCF/PA11 72/28	0°	8	57.77 ± 4.23	920.0 ± 40.7	1.69 ± 0.10
rCF/PA11 80/20	0°	10	64.31 ± 6.28	814.6 ± 85.6	1.61 ± 0.24
rCF/PA6 70/30	0°	9	83.89 ± 10.36	1187.9 ± 68.0	1.08 ± 0.44
rCF/PA11 72/28	90°	1	2.70 *	20.7 *	2.11 *
rCF/PA11F 80/20	90°	2	2.72 ± 0.22	18.0 ± 0.1	1.22 ± 0.13
rCF/PA6 70/30	90°	2	4.36 ± 0.05	31.3 ± 0.2	1.11 ± 0.02

* The supplied campaign contained one valid representative transverse rCF/PA11 72/28 curve. It is noted that, in Table 3, values are mean ± standard deviation for the stated valid *n*, except for the 72/28 transverse result (*n* = 1), which is an individual observation.

**Table 4:** Comparison of yarn- and tape-derived PA11/rCF laminates at comparable fiber volume content.

Architecture	FVC [vol.%]	Axial Modulus, E_11_ [GPa]	Axial Strength, σ_11_ [MPa]	Transverse Modulus, E_22_ [GPa]	Transverse Strength, σ_22_ [MPa]	Shear Modulus, G_12_ [GPa]	Shear Strength, τ_12_ [MPa]
Published rCF/PA11 tape 70/30 [25]	56.72	48.14 ± 4.55	866.2 ± 127.2	3.91 ± 0.21	52.9 ± 2.0	3.56 ± 0.58	116.5 ± 0.7
rCF/PA11yarn 72/28	54.39	57.77 ± 4.23	920.0 ± 40.7	2.70 *	20.7 *	1.87 ± 0.03	48.7 ± 1.4
rCF/PA11 yarn 80/20	58.51	64.31 ± 6.28	814.6 ± 85.6	2.72 ± 0.22	18.0 ± 0.1	—	—

Tape values and their reported scatter are taken from [25]. * The yarn 72/28 transverse values are from one valid specimen (*n* = 1). A dash denotes a property not tested.

**Table 5:** Mechanical performance of the connecting-rod-shaped structural benchmark configurations.

Material/configuration	*n*	Thickness [mm]	Mass [g]	*F*_max_ [kN]	*F*_max_/*m* [kN/g] *	*F*_max_/*S*_0_ [MPa]
rCF/PA11 70/30, unsupported	4	2.23 ± 0.01	22.3 ± 1.0	7.9 ± 0.3	0.354	78.8
rCF/PA11 70/30, original plate	2	2.19 ± 0.07	22.5 ± 0.7	7.8 ± 0.2	0.347	79.2
rCF/PA11 70/30 + M24 washers	2	2.53 ± 0.16	25.5 ± 2.1	11.2 ± 0.6	0.439	98.5
rCF/PA11 72/28, supported	3	2.20 ± 0.06	23.0 ± 1.0	11.3 ± 0.5	0.491	114.1
Virgin-CF/PA12, 2.1 mm	2	2.06 ± 0.11	19.5 ± 0.7	9.0 ± 0.4	0.462	97.3
Virgin-CF/PA12, 2.7 mm	2	2.72 ± 0.05	28.0 ± 0.0	16.1 ± 1.1	0.575	131.7
CF/epoxy RTM	5	1.57 ± 0.01	16.2 ± 0.4	8.4 ± 0.4	0.519	118.6
Aluminum	3	1.90 ± 0.02	37.3 ± 0.6	10.7 ± 0.2	0.287	125.1

* Nominal stress was calculated using the measured thickness and nominal width. It is a structural-coupon metric affected by hole bearing and support conditions, not an intrinsic tensile strength.

**Table 6:** Screening climate-change inventory and total impact per benchmark part *.

Route	Fiber [kg CO_2_-eq]	Matrix [kg CO_2_-eq]	Yarn [kg CO_2_-eq]	TFP [kg CO_2_-eq]	Compression Molding [kg CO_2_-eq]	RTM [kg CO_2_-eq]	Total [kg CO_2_-eq]
rCF/PA11	0.0755	0.0417	0.0184	0.0180	0.2636	0.0000	0.4172
vCF/PA12	0.6809	0.1464	0.0187	0.0180	0.2636	0.0000	1.1275
rCF/PA6	0.0597	0.1522	0.0192	0.0180	0.2636	0.0000	0.5126
vCF/Epoxy	0.6627	0.1709	0.0192	0.0090	0.0000	0.0084	0.8703

* For rCF routes, “Fiber” denotes the recycled-fiber contribution. The values are based on the screening inventory and should be interpreted under the methodological assumptions and limitations described in Section 2.7.

## Data Availability

The processed data supporting the findings are provided in the tables and figures. Raw mechanical, textile-processing, and life-cycle inventory data are available from the corresponding author subject to the confidentiality agreements of the project partners.
